# Functional Significance of Satellite DNAs: Insights From *Drosophila*

**DOI:** 10.3389/fcell.2020.00312

**Published:** 2020-05-05

**Authors:** Aleksei S. Shatskikh, Alexei A. Kotov, Vladimir E. Adashev, Sergei S. Bazylev, Ludmila V. Olenina

**Affiliations:** ^1^Laboratory of Analysis of Clinical and Model Tumor Pathologies on the Organismal Level, Institute of Molecular Genetics, Russian Academy of Sciences, Moscow, Russia; ^2^Laboratory of Biochemical Genetics of Animals, Institute of Molecular Genetics, Russian Academy of Sciences, Moscow, Russia

**Keywords:** satellites, *Drosophila*, segregation distortion, meiotic drive, heterochromatin, centromere, chromosome segregation, reproductive isolation

## Abstract

Since their discovery more than 60 years ago, satellite repeats are still one of the most enigmatic parts of eukaryotic genomes. Being non-coding DNA, satellites were earlier considered to be non-functional “junk,” but recently this concept has been extensively revised. Satellite DNA contributes to the essential processes of formation of crucial chromosome structures, heterochromatin establishment, dosage compensation, reproductive isolation, genome stability and development. Genomic abundance of satellites is under stabilizing selection owing of their role in the maintenance of vital regions of the genome – centromeres, pericentromeric regions, and telomeres. Many satellites are transcribed with the generation of long or small non-coding RNAs. Misregulation of their expression is found to lead to various defects in the maintenance of genomic architecture, chromosome segregation and gametogenesis. This review summarizes our current knowledge concerning satellite functions, the mechanisms of regulation and evolution of satellites, focusing on recent findings in *Drosophila*. We discuss here experimental and bioinformatics data obtained in *Drosophila* in recent years, suggesting relevance of our analysis to a wide range of eukaryotic organisms.

## Introduction

It is established that genome sizes do not correlate with the complexity of eukaryotic organisms; this phenomenon is known as the C-value paradox (Doolittle, 2013; Graur et al., 2013). The number of protein-coding genes in species with various biological complexities may differ insignificantly (approximately 20.2 thousand genes in *Caenorhabditis elegans*, 14 thousand in *Drosophila melanogaster* and 20.4 thousand in *Homo sapiens*, according to Ensembl Genome Browser data). Eukaryotic genomes often contain vastly different amounts of total DNA, and most of their DNA does not encode proteins (Elliott and Gregory, 2015). Because this non-coding DNA did not associate with any protein-coding functions, historically it was considered as non-functional “junk” or parasitic DNA (Ohno, 1972). Over the years, the concept of the uselessness of non-coding DNA has been constantly revised and has undergone significant changes to date owing to the extensive characterization of this genomic portion (Csink and Henikoff, 1998; Pennisi, 2012; Lower et al., 2018). Extended non-coding regions of the genome are present in all eukaryotes and persist across the evolutionary history of individual species both in the conservative and non-conservative modes (Plohl et al., 2012; Harmston et al., 2013; Polychronopoulos et al., 2017). Satellites are multi-copy tandemly repeated DNA sequences that constitute the largest part of the non-coding genomes of eukaryotes (Lopez-Flores and Garrido-Ramos, 2012; Biscotti et al., 2015). Satellite repeats possess extreme diversity in their monomer size, nucleotide sequence, complexity, genomic distribution, and abundance even in closely related species (Lohe and Brutlag, 1987; Plohl et al., 2012).

A common property of different satellite repeats is their heterochromatic state. The term “satellite DNA” originally referred to a fraction of total genomic DNA which formed additional bands in CsCl gradients, due to its unusual nucleotide composition and floating density (Kit, 1961). It was later established that this fraction is heterogeneous and, besides satellite repeats itself, also contained sequences of different nature, including transposable elements and some multi-copy gene families such as ribosomal DNA and histone genes. Satellite DNA forms essential structures of chromosomes, such as telomeres and centromeres, ensuring protection and stability of chromatin of these sites (Yunis and Yasmineh, 1971; Garrido-Ramos, 2017; Lower et al., 2018). At the same time, satellites are highly variable in their sequence and in the number of copies both within a species and between closely related species that indicates rapid evolution of satellites (Lohe and Brutlag, 1987; Plohl et al., 2012; Larracuente, 2014; Jagannathan et al., 2017). Satellite arrays as genomic structures contribute to multiple cellular processes, such as proper chromosome segregation in the cell cycle, regulation of gene expression, and genome stability maintenance. Many satellites are transcribed producing non-coding RNAs (Ugarkovic, 2005; Biscotti et al., 2015; Ferreira et al., 2015; Kuhn, 2015). Misregulation of their expression leads to various developmental defects in the maintenance of genomic architecture, chromosome segregation, and gametogenesis. Disorders associated with satellites may be linked with a number of human diseases, such as hereditary diseases, developmental abnormalities, different cancers, neurodegenerative disorders, aneuploidy, and others (Bersani et al., 2015; Scacheri and Scacheri, 2015; Zhang and Lupski, 2015; Aldrup-MacDonald et al., 2016). Variations in satellite repeat abundance are also found to be associated with evolution of genome structure (Charlesworth et al., 1994), hybrid incompatibility and speciation (Ferree and Barbash, 2009), dosage compensation (Joshi and Meller, 2017) and meiotic drive (Henikoff et al., 2001; Fishman and Saunders, 2008; Larracuente and Presgraves, 2012). However, despite intensive investigations, many functions of these non-coding genomic elements are still largely obscure.

In this review, we focus on the current advances in the understanding of satellite functions in eukaryotic genomes, generally restricting our viewing to recent findings in *Drosophila*. Experimental data obtained in recent years using such a valuable model organism as *Drosophila*, as well as the emergence of high-throughput technologies in sequencing, assembly and analysis of heterochromatic genomic regions provide significant insight into the mechanisms of regulation and evolution of satellites and allow deciphering their non-obvious biological properties.

## General Properties of Satellites in the *Drosophila* Genome

### Structure of Satellite DNA

Satellites can be classified by the length of their repeating units: in addition to satellites consisting of long monomers from a hundred to several hundred nucleotides, minisatellites (from 10 to 100 bp) and microsatellites (from one to 10 bp) are also distinguished (Ramel, 1997; Lopez-Flores and Garrido-Ramos, 2012; Garrido-Ramos, 2017). Several families of satellites can be simultaneously present in the genome of one species, and more than 17 different satellite types were found in the *D. melanogaster* genome (Lohe et al., 1993; Jagannathan et al., 2017; Talbert et al., 2018; Chang and Larracuente, 2019; Chang et al., 2019) (Table 1), whereas only 9 families of satellite DNA were identified within the human genome (Levy et al., 2007; Miga, 2015). *D. melanogaster* appears to be an ideal model to investigate the genomic organization and distribution of satellites, as it has a relatively small genome (approximately 180 Mb), organized in just three autosomes, chromosomes 2, 3, and 4, and two sex chromosomes. In *D. melanogaster*, the most abundant satellites are very short 5- to 12-bp repeats, with the largest fraction being represented by (AAGAG)_n_ repeats (Lohe et al., 1993; Tolchkov et al., 2000; Torok et al., 2000; Jagannathan et al., 2017; Talbert et al., 2018). Along with satellites consisting of short repeating units, the *D. melanogaster* genome also contains satellites with long repeating sequences, such as the intergenic spacer of ribosomal genes (*IGS*), the 1.688 family of repeats, and *Rsp* repeats containing multiple tandem dimers of the left and right 120-bp sequences (Table 1). The most abundant satellite family of the human genome is alpha satellite DNA with a monomer unit of about 170 bp, representing more than 50% of the total content of satellite DNA in humans (Levy et al., 2007; Miga, 2015). At the same time, total content of satellite DNA in different species varies from less than one percent to more than a half of the genome. Even closely related species can differ greatly in their satellite content: satellite DNA composes only 0.5% of the *Drosophila erecta* genome, whereas *Drosophila simulans* has 5%, *D. melanogaster* has over 20%, and *Drosophila virilis* has nearly 50% of satellite DNA in the genome (Wei et al., 2014; Garrido-Ramos, 2017; Jagannathan et al., 2017; Flynn et al., 2020).

**TABLE 1:** Distribution of the most represented satellite repeats of *Drosophila melanogaster* by chromosomes.

**Satellite**	**Location**	**References**
(TAGA)_n_	X	Jagannathan et al., 2017
(AAAAC)_n_	Y	Jagannathan et al., 2017
(AAGAC)_n_	Y, 2	Lohe et al., 1993; Jagannathan et al., 2017; Chang and Larracuente, 2019
(AACAC)_n_	Y, 2	Jagannathan et al., 2017; Chang and Larracuente, 2019
(AAGAG)_n_	All	Lohe et al., 1993; Jagannathan et al., 2017; Chang and Larracuente, 2019;
(AATAC)_n_	Y	Lohe et al., 1993; Jagannathan et al., 2017
(AATAG)_n_	Y, 2, 3	Lohe et al., 1993; Jagannathan et al., 2017; Chang and Larracuente, 2019
(AATAT)_n_	All	Lohe et al., 1993; Tolchkov et al., 2000; Jagannathan et al., 2017
(AAGAGAG)_n_	Y, 2	Lohe et al., 1993; Jagannathan et al., 2017; Chang and Larracuente, 2019
(AACAAAC)_n_	2	Jagannathan et al., 2017
(AATAAAC)_n_	Y	Jagannathan et al., 2017
(AATAGAC)_n_	Y	Jagannathan et al., 2017; Chang and Larracuente, 2019
*Prodsat* (AATAACATAG)_n_	2, 3	Lohe et al., 1993; Torok et al., 2000; Garavis et al., 2015b; Jagannathan et al., 2017
*dodeca* (CGGTCCCGTACT/GGTCCCGTACT)_n_	3	Lohe et al., 1993; Garavis et al., 2015b; Jagannathan et al., 2017
*IGS* (intergenic spacer) (240-bp)	X, Y, 3	Jagannathan et al., 2017; Chang et al., 2019; Chang and Larracuente, 2019
1.688 family (353/356/359/372/260-bp)	X, 3 2 (260-bp)	Lohe et al., 1993; Tolchkov et al., 2000; Jagannathan et al., 2017
*Rsp* (120-bp + 120-bp)	2, 3	Houtchens and Lyttle, 2003; Larracuente, 2014; Khost et al., 2017

### Satellite Arrays as Platforms for Centromeric and Pericentromeric Regions of Genome

Satellite repeats are predominantly located in centromeric and pericentromeric regions of chromosomes. Centromeres are chromosomal sites for assembly of kinetochores, protein complexes that attach to spindle fibers and mediate separation of chromosomes during cell division. While pericentromeric regions possess typical heterochromatic structure, centromeric chromatin is different from both euchromatin and the surrounding pericentromeric heterochromatin. The key epigenetic determinant of the centromere is a special variant of histone H3, CENP-A (CID in *Drosophila*), which in complex with canonical histones H2A, H2B, and H4 forms specialized nucleosomes from centromeric DNA and recruits kinetochore subunits and spindle assembly checkpoint proteins to the centromere (Allshire and Karpen, 2008; Tachiwana and Kurumizaka, 2011; Steiner and Henikoff, 2015; Barra and Fachinetti, 2018; Smurova and De Wulf, 2018). CENP-A interacts with CENP-C, one of the key factors of kinetochore assembly (Przewloka et al., 2011), and usually marks active centromeres independently of their DNA sequence (Kato et al., 2013). Centromeric chromatin is devoid of typical heterochromatin marks and contains active histone modifications, such as H3K4me1, H3K4me2, H3K36me2, and H3K36me3, inherent for euchromatin. However, unlike typical euchromatin, centromeric chromatin does not contain acetylated histones (Sullivan and Karpen, 2004). In accordance with the current view, most animals and plants have a similar organization of centromeres: they contain megabase arrays of tandem satellite repeats as major structural elements; and a single satellite family typically prevails at the centromeres of all chromosomes of a species (Allshire and Karpen, 2008; Henikoff et al., 2015; McKinley and Cheeseman, 2016). However, our understanding of centromere structure has been based predominantly on cytological studies. Centromeres are embedded in expanded satellite-rich pericentromeric regions, and to date their endogenous sequences have been largely absent in the complete genome assemblies, owing to intrinsic problems in the assembly of long stretches of tandem repeats (Chang et al., 2019).

Recently the organization of all functional centromeres of *D. melanogaster* has been resolved in detail for the first time, owing to the mapping of CENP-A-occupied regions using the ChIP-seq approach and *de novo* chromatin assembly methods. It is shown that CENP-A occupies islands of complex DNA, which are composed of retrotransposons such as *G2/Jockey-3*, *Doc*, and *Doc-2* flanked by large blocks of satellite repeats (Chang et al., 2019). *Dodeca* satellite and its variants, tandem (AATAG)_n_ and (AATAT)_n_ repeats, and also *Prodsat* (*Prod* satellite; also known as the 10-bp satellite) are shown to make up the majority of centromeric tandem repeats (Talbert et al., 2018; Chang et al., 2019) (Table 2). Note, that the centromere of chromosome 3 contains about 240 copies of the centromere-specific variant of the *IGS* repeats and also an expanded array of the *dodeca* satellite with small insertions of retrotransposons and DNA transposons (Chang et al., 2019). Surprisingly, satellites are practically not represented in the centromere of the Y chromosome, despite the fact that the whole Y chromosome is enriched in tandem repeats (Chang and Larracuente, 2019; Chang et al., 2019) (Tables 1, 2). None of the sequences contained within the centromeres are found to be exclusive to centromeres. Each centromere has a unique structure, although many of their constituent elements are common among them. Specifically, all centromeres of *D. melanogaster* are enriched with non-LTR retroelement *G2/Jockey-3*, which is also found in the centromeres of the sibling species *D. simulans* (Chang et al., 2019), revealing striking conservation between these species despite the divergence in their centromeric satellite DNAs (Talbert et al., 2018). Retrotransposons are also found at the centromeres from fungi to humans, indicating that they appear to be common centromeric components (Miga et al., 2014; Yadav et al., 2018).

**TABLE 2:** Location of satellites in centromeric and pericentromeric regions of the *D. melanogaster* genome.

**Chromosome**	**Pericentromeric satellites**	**Centromeric satellites**
X	(AAGAT)_n_, 359-bp (1.688 family)	(AAGAG)_n_, (AATAT)_n_
Y	(AATAT)_n_	–
2	*Prodsat* (AATAACATAG)_n_, 260-bp (1.688 family), *Rsp*	(AAGAG)_n_, (AATAG)_n_
3	*Prodsat* (AATAACATAG)_n_, (AATAG)_n_, 353-bp and 356-bp (1.688 family)	*IGS, dodeca* (CGGTCCCGTACT/GGTCCCGTACT)_n_, *Prodsat* (AATAACATAG)_n_
4	(AAGAG)_n_	(AAGAT)_n_

**Presented data are based on the analysis of the following publications Lohe et al., 1993; Larracuente, 2014; Garavis et al., 2015b; Jagannathan et al., 2017; Khost et al., 2017; Talbert et al., 2018; Chang et al., 2019.**

Centromeric DNA sequences themselves are not sufficient to specify centromere position. It has been proposed that centromeres are epigenetically identified, and satellite DNAs appear to function in establishing the corresponding epigenetic landscape, recruiting specific protein complexes and thus contributing to the formation of centromere-specific chromatin (Allshire and Karpen, 2008; Westhorpe and Straight, 2014; McKinley and Cheeseman, 2016). The epigenetic hypothesis of centromere specification is supported by the existence of neo-centromeres, which can arise ectopically in *D. melanogaster* (and in many other species, including humans), upon γ-irradiation-induced chromosome breakage or overexpression of CID (Maggert and Karpen, 2001; Olszak et al., 2011; Burrack and Berman, 2012). However, recently it is found that centromeric satellite DNAs may fold into specific cubic-like higher-ordered structures, which are capable of attracting specific nuclear proteins. The capacity of *dodeca* satellite repeats of *D. melanogaster* to adopt these secondary structures is proven by NMR, circular dichroism and mass spectrometry methods (Garavis et al., 2015b). This pioneering study shows that the C-rich strand of *dodeca* satellite is able to form dimeric i-motif structures *in vitro*, defined as cubic-like four-stranded DNA structures generated by the association of two parallel DNA duplexes combined in an antiparallel manner. A megabase array of *dodeca* satellite repeats is found in the centromere of chromosome 3; however, likely a part of them extends beyond the centromeric chromatin to the right arm (Garavis et al., 2015b; Jagannathan et al., 2017; Chang et al., 2019). Similarly, human centromeric alpha satellites and murine centromeric Y-satellites are also found to generate dimeric i-motifs (Garavis et al., 2015a). Subsequent studies have shown that centromeric satellites in a wide range of animals can shape different non-canonical secondary structures, including single-stranded DNA, hairpins, and R-loops (Kabeche et al., 2018; Kasinathan and Henikoff, 2018).

Taken together, these data indicate that functional centromeres of eukaryotes adopt specific secondary structures, which facilitate their identification as specialized chromosome regions. In primates, including humans, sequence-specific sites also facilitate the assembly of centromeric chromatin. The monomers of centromeric alpha satellites contain a specific 17-bp protein-binding motif called CENP-B box which binds CENP-B protein (Haaf et al., 1995; Ohzeki et al., 2002; Masumoto et al., 2004). It has been shown that the binding of CENP-B to DNA promotes *de novo* formation of CENP-A-containing centromeric chromatin (Okada et al., 2007). CENP-B-like boxes are also found in unrelated centromere satellites of root-knot nematodes (Meštrović et al., 2013), in the mouse (Broccoli et al., 1990), and Antarctic scallop (Canapa et al., 2000). In other species, including *Drosophila*, instances of sequence-specific formation of centromeric chromatin are not found. Thus, centromere specification may be based on both structural motifs in centromeric DNA and epigenetic mechanisms; they putatively function in concert ensuring proper centromere formation.

The functions of pericentromeric satellite DNAs are still poorly understood. Pericentromeric regions possess classic epigenetic marks of constitutive heterochromatin such as H3K9me2, H3K9me3, and H3K27me3, and H4K20me3 (Richards and Elgin, 2002; Nishibuchi and Dejardin, 2017). It is proposed that satellite repeats are necessary for the formation and maintenance of pericentromeric heterochromatin, which contributes to repression of transposable elements, ensuring genome stability. In addition, pericentromeric regions contain a number of genes whose expression requires a heterochromatic environment (Yasuhara and Wakimoto, 2006). Pericentromeric heterochromatin is found to be involved in the stabilization of the cohesin — a protein complex that ensures sister chromatid cohesion (Bernard et al., 2001; Nasmyth and Haering, 2009; Ng et al., 2009). Heterochromatin establishment in *Drosophila* early embryogenesis is characterized by the formation of a densely packed chromatin structure, called the chromocenter, during mitotic divisions 9–10 (Pimpinelli et al., 1985; Kellum et al., 1995). More than 50 years ago the association of pericentromeric satellite DNAs of heterologous chromosomes into the chromocenter within interphase nuclei was revealed at the cytological level (Jones, 1970; Pardue and Gall, 1970). Recently it is found that pericentromeric satellite sequences interact with AT-hook DNA-binding proteins, such as D1 in *D. melanogaster* and HMGA1 in mouse (Jagannathan et al., 2018). These proteins facilitate the bundling of satellite DNAs of multiple chromosomes to form a single chromocenter in many types of cells. The generation of the chromocenter facilitates nuclear assembly after cell division preventing individual chromosomes from floating out of the nucleus with the formation of non-functional micronuclei and cell death (Jagannathan et al., 2018). D1 and Proliferation disrupter (Prod) DNA-binding protein sequence-specifically recognize (AATAT)_n_ satellites on the chromosome 4 and sex chromosomes and *Prod* satellites on the chromosomes 2 and 3, respectively. D1 and Prod are found to transiently interact with each other in interphase nuclei providing the incorporation of the chromosomes in the single chromocenter. Double mutants of *D1* and *prod* cause an increase in micronuclei generation and lead to strong embryonic lethality indicating the essential function of chromocenters and satellite DNAs for cell viability. According to these data, the chromocenter is a structure consists of dynamically interacted modules of satellites and satellite-binding proteins (Jagannathan et al., 2019). Based on these results, a new model for functions of pericentromeric satellite DNA has been proposed. Pericentromeric satellites (Table 2) create the architectural platform for the association of heterologous chromosomes in the single chromocenter. Thus, satellite DNAs serve as critical regions of eukaryotic chromosomes ensuring correct encapsulation of all chromosomes in the interphase nucleus. It is essential that satellite DNAs with different sequences are bound by proteins capable of bundling multiple chromosomes. In accordance with these findings, chromocenter bundling proteins and pericentromeric satellite sequences appear to be co-evolving (Jagannathan et al., 2018, 2019). This leads to the accumulation of long stretches of repetitive sequences, which presumably form a unique DNA architecture for recognition by DNA-binding proteins.

### Transcription of Satellites and Its Functional Significance

Since satellite DNAs do not contain ORFs and are generally characterized by a tightly packed heterochromatic structure in the genome, earlier it was believed that they are not transcribed and do not have biological functions that fits into the paradigm of “junk” DNA. Although transcription of satellite DNA was first described more than 50 years ago (Harel et al., 1968; Cohen et al., 1973), these data were not widely accepted for a long time. However, subsequent studies revealed that transcription of satellites can be considered a common property of eukaryotic genomes. Expression of satellite DNA appears to be temporarily and developmentally regulated in certain cells and tissues under normal conditions and also in response to stress (Ugarkovic, 2005; Biscotti et al., 2015; Ferreira et al., 2015; Kuhn, 2015). Dynamics of pericentromeric heterochromatin formation in higher eukaryotes is now actively investigated, and current data support a role of satellite DNA transcription in heterochromatinization of pericentromeric regions (Bernstein and Allis, 2005; Chan and Wong, 2012; Shatskikh and Gvozdev, 2013).

**FIGURE 1:**
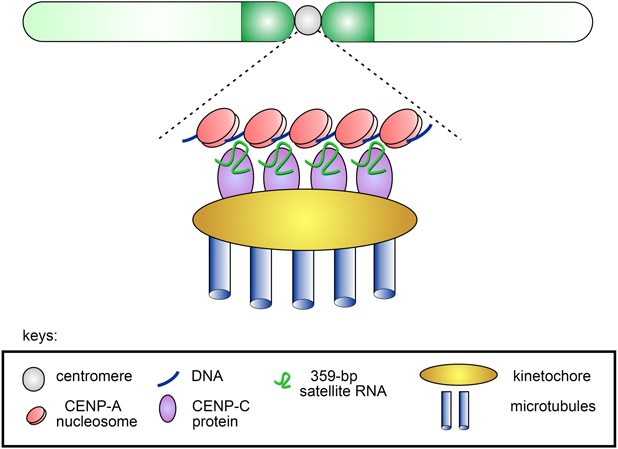
Scheme of the putative contribution of 359-bp satellite RNAs to CENP-A and CENP-C deposition at the centromeres of *Drosophila melanogaster*. The stable association of CENP-C and CENP-A with the centromere ensured by the presence of satellite transcripts leads to the formation of a functional kinetochore and subsequent accurate segregation of chromosomes in mitosis.

It was initially assumed that satellite transcripts could result from read-through transcription from promoters of adjacent active genes (Diaz et al., 1981). However, subsequently binding sites for transcription factors and active promoters recruiting PolII or PolIII, as well as other functional elements, were found inside satellite repeats themselves (Csink and Henikoff, 1998; Ugarkovic, 2005; Salvany et al., 2009). Transcription factor GAGA is associated with GA-rich satellite repeats (AAGAG)_n_ and (AAGAGAG)_n_ in multiple regions of the *D. melanogaster* genome (Raff et al., 1994; Platero et al., 1998) and may contribute to transcription of satellite DNAs. Transcription of satellite DNAs is differentially regulated throughout the cell cycle and during ontogenesis and is found to be essential for development (Probst et al., 2010). Satellite DNAs are generally transcribed as long or small non-coding transcripts. Transcripts of satellite DNAs differ in length and strand specificity, some transcripts are polyadenylated and exported to the cytoplasm, while others are found only in the nuclei, like (AAGAC)_n_ repeats encoded by Y-chromosomal loops and expressed specifically in spermatocytes of *D. melanogaster* and *Drosophila hydei* (Trapitz et al., 1988; Bonaccorsi et al., 1990; Fingerhut et al., 2019), as mentioned below. X-specific pericentromeric 359-bp repeats of the 1.688 family are transcribed in both sense and antisense orientation in S2 cell culture and embryos with the formation of long non-coding transcripts of different sizes varying from one to up to four repeating units (Salvany et al., 2009; Rošić et al., 2014). These transcripts are localized within the nuclei throughout the cell cycle and are found at the centromeres of the X chromosome and major autosomes during mitosis. Transcription of the 359-bp repeats depends on homeobox-containing transcription factor Homothorax (Hth), and *hth* mutations cause abnormal distribution of CENP-A (Salvany et al., 2009). Depletion of RNAs transcribed from the 359-bp repeats leads to mitotic defects in anaphase of S2 cells characterized by lagging of all major chromosomes, which do not segregate properly. About half of the cells with lagging chromosomes subsequently generated micronuclei and died. It is found that 359-bp satellite RNAs interact with the centromeric protein CENP-C and this interaction mediates localization of the satellite RNAs near centromeres in mitosis. Upon CENP-C depletion, 359-bp satellite RNAs are delocalized from centromere regions. In turn, knockdown of 359-bp repeat RNAs leads to reduction of newly synthesized CENP-C and CENP-A at the centromeres. This mediates a negative effect on the attraction of kinetochore proteins to the centromeres during mitosis that subsequently causes chromosome segregation defects and genome instability (Rošić et al., 2014). Thus, non-coding transcripts of 359-bp satellites contribute to the safeguard mechanism that is required for supporting CENP-A deposition at the centromeres and correct chromosome segregation (Figure 1). Whereas the paper of Rošić et al. (2014) described that X-specific 359-bp repeat transcripts function *in trans* at the centromeres of autosomes, recently it is found that these transcripts can be produced during mitosis by local transcription from the centromeric DNA of autosomes (Bobkov et al., 2018). This finding is in agreement with recently published data that transcripts of human alpha satellites involved in CENP-A loading are also produced *in cis* at centromeres (McNulty et al., 2017). However, it should be noted that 359-bp repeats do not constitute a significant part of centromeric or pericentromeric DNA sequences of *Drosophila* autosomes (Chang et al., 2019; Table 2). Therefore, further studies are needed to elucidate the question about the functioning of these satellite transcripts predominantly *in cis* or *in trans*.

Three Y chromosome lampbrush-like loops that are visible in primary spermatocyte nuclei of *D. melanogaster* outside the X-Y chromosome territory have been known for the last 50 years (Meyer et al., 1961). However, the functional significance of Y-loop formation has been enigmatic. Only a few genes reside in the Y chromosome, and among them there are six genes encoding the so-called “fertility factors” (*kl-1, kl-2, kl-5, ks-1, ks-2*, and *kl-3*) (Hardy et al., 1981; Carvalho et al., 2000). Strikingly, three of them, *kl-5, kl-3*, and *ks-1*, contain gigantic megabase-sized introns filled with simple satellite repeats (AAGAC)_n_ and (AATAT)_n_, comprising more than 99% of the corresponding gene loci. It was shown that these genomic regions form lampbrush-like nucleoplasmic structures in spermatocytes, named A, B and C Y-loops (Bonaccorsi et al., 1998). The existence of Y-loop structures reflects specific transcription of the underlying genes in spermatogenesis that has been found to be a conserved feature across the *Drosophila* genus, including *D. melanogaster, D. simulans, Drosophila yakuba, Drosophila pseudoobscura, D. hydei*, and *Drosophila littoralis* (Hess, 1967; Piergentili, 2007; Fingerhut et al., 2019). Transcription of Y-loop regions as single transcripts appears to take place in the *D. melanogaster* testes during 90-h stage of primary spermatocyte maturation. Two RNA-binding factors, Blanks and Hephaestus, are found to localize to Y-loops and are required for transcription or proper processing of the Y-loop gene transcripts. The corresponding *blanks* or *hephaestus* mutations lead to male sterility owing to defects in sperm individualization, reflecting the phenotypes observed in males with knockdowns of *kl-5* and *kl-3* genes encoding axonemal dyneins (Fingerhut et al., 2019). Although the functional relevance of the gigantic intron sequences still remains obscure, intron size and content could play a critical role in the regulation of expression of the Y-loop genes. The satellite repeats inside the introns could allow the recruitment of the transcriptional machinery to the Y-loops to enhance their expression, providing an example of a unique program of transcriptional regulation.

In recent studies it is shown that (AAGAG)_n_ satellite RNAs are transcribed from heterochromatic regions of multiple chromosomes of *D. melanogaster* throughout a wide developmental period from embryos to adulthood producing a novel class of long non-coding RNAs (Pathak et al., 2013; Mills et al., 2019). Transcripts of (AAGAG)_n_ repeats are found to be an essential component of the nuclear matrix (Pathak et al., 2013). (AAGAG)_n_ transcripts are also maternally inherited as long non-coding RNAs, which are specifically colocalized with H3K9me3 foci in the embryonic nuclei since heterochromatin formation at cycle 14. Depletion of (AAGAG)_n_ RNA causes a loss of the sub-nuclear structural integrity and results in a significantly lower fly viability at larval and pupal stages (Pathak et al., 2013; Mills et al., 2019). In the testes of adult males, high levels of (AAGAG)_n_ RNA expression are found in primary spermatocytes, where these transcripts are enriched in regions adjacent to DAPI-stained chromatin near the nuclear periphery. Expression of (AAGAG)_n_ RNA in primary spermatocytes is essential for male fertility: depletion of (AAGAG)_n_ RNA in the germline causes complete male sterility, with no impact on female fertility. (AAGAG)_n_ RNA depletion leads to aberrant spermatid individualization process displaying lagging of spermatid nuclei and loosely packed spermatid bundles, and causes reduced and defective incorporation of transition protein Mst77F in spermatid DNA, and subsequent failure in histone-protamine transitions (Mills et al., 2019). It should be noted that expression of (AAGAG)_n_ RNA in primary spermatocytes is necessary for promoting sperm development at much later post-meiotic stages of spermatogenesis. We found that in the male germline, transcripts of (AAGAG)_n_ repeats undergo subsequent processing with the generation of 23–29 bp small RNAs, presumably piRNAs, however, their role in male fertility is needed to elucidate yet.

Small RNAs generated by the processing of satellite transcripts by the RNAi machinery are found to date in several species, including *Drosophila*. The RNAi pathways and small RNAs are known to be involved in the establishment and maintenance of pericentromeric heterochromatin (Bernstein and Allis, 2005; Ferreira et al., 2015). Autosomal satellites of the 1.688 family (361-bp and 260-bp) are bi-directionally transcribed in the ovarian germline of *D. melanogaster* with subsequent generation of germline-specific small piRNAs from long RNA precursors (Usakin et al., 2007); however, their exact functions in oogenesis are not clear to date. Transcription and generation of small RNAs from satellites are found be involved in dosage compensation and meiotic drive, as discussed below.

Thus, satellite transcription and generation of satellite-derived small RNAs appear to be necessary for fly viability and fertility, the formation of heterochromatin domains, centromere function and proper chromosome segregation, chromatin-mediated regulation of gene expression. As described in the next section satellite-derived small RNAs also contribute to facilitating dosage compensation.

## Satellites and Dosage Compensation

Males of numerous species, including humans and *Drosophila*, are heterogametic and carry a gene-rich X chromosome and an almost completely heterochromatic Y chromosome with few protein-coding genes. The problem of the difference in the sex chromosomes is resolved by dosage compensation to ensure an equal level of expression of X-linked genes in males and females and prevent functional aneuploidy in males. Although such compensation is achieved by several distinct ways in different species, it always requires coordinated regulation of the entire X chromosome (Disteche, 2016; Kuroda et al., 2016). The underlying mechanisms include up-regulation of the X chromosome in the heterogametic sex, as in *Drosophila*, or random inactivation of one of the X chromosomes in the homogametic sex, as in mammals.

It has been shown that in somatic cells of *D. melanogaster* males most X-linked genes show an approximately twofold increase in their expression (Prestel et al., 2010; Lucchesi and Kuroda, 2015). A key component of the dosage compensation system in *Drosophila* is the male-specific lethal (MSL) complex. It is a ribonucleoprotein complex of five proteins MLE, MSL1, MSL2, MSL3, MOF, and two long non-coding RNAs, *roX1* and *roX2*, with redundant, but essential functions (Meller and Rattner, 2002; Deng and Meller, 2006; Conrad and Akhtar, 2012; Koya and Meller, 2015). Since one of the key components of MSL, MSL2, undergoes translational repression in females, the MSL complex is not assembled there. MSL-mediated dosage compensation initiates in developing male embryos at 3 h after egg deposition. The MSL complex is selectively enriched on the single male X chromosome, where it provides chromatin modification via histone H4 acetylation at lysine 16 (H4K16ac) by acetyltransferase MOF (Hallacli and Akhtar, 2009). Hyperacetylation of X-linked chromatin at H4K16 leads to relaxed chromatin structure, improved accessibility for transcription factors from the nucleoplasm, and facilitated progression of Pol II through transcribed gene regions (Bell et al., 2010; Larschan et al., 2011; Conrad and Akhtar, 2012; Ferrari et al., 2013).

Prominent molecular markers of the male X chromosome are non-coding transcripts *roX1* and *roX2.* Both *roXs* are encoded on the X chromosome. Regions of *roX* RNA transcription are considered sites of the co-transcriptional assembly of the MSL complex (Kelley et al., 1999; Park et al., 2002). The next step includes recruitment of the assembled MSL complexes to X-linked chromatin entry sites (CESs), containing 21-nt GA-rich motifs called MSL Recognition Elements (MREs) (Alekseyenko et al., 2008; Straub et al., 2008). Adaptor protein CLAMP binds to MREs and attracts to them the completely assembled MSL complex (Soruco et al., 2013). Then MSL appears to spread in *cis* into neighboring actively transcribed genes (Straub and Becker, 2007). Strikingly, MREs are found to be functionally conserved in *Drosophila miranda* (Alekseyenko et al., 2013), a species which diverged from *D. melanogaster* about 50 MYA.

However, MREs alone cannot be responsible for specific X chromatin targeting, because MREs are only two-fold enriched on the X chromosome compared to the autosomes (Alekseyenko et al., 2008). It is shown that autosomal MREs also recruit CLAMP, but fail to recruit the MSL complex to themselves. Despite more than 25 years since MSL has been visualized at hundreds of sites along the whole X chromosome in males (Kuroda et al., 1991), the mechanism of selective MSL X-targeting is not clearly understood yet (Conrad and Akhtar, 2012; Gallach, 2015).

Studies performed in the Meller lab revealed a potential role of siRNAs in this process. These siRNAs are generated at early embryonic stages from a subset of X-linked euchromatic 359-bp repeats that are members of the 1.688 satellite family (designated here and after as 1.688^X^) (Menon et al., 2014; Joshi and Meller, 2017). Hundreds of 1.688^X^ repeats grouped in short arrays are specifically enriched along the entire length of the X chromosome, including regions of active transcription and gene introns (Kuhn et al., 2012). 1.688^X^ repeats possess, on average, 73% sequence identity among themselves (Kuhn et al., 2012). The enrichment of the X euchromatin in satellite repeats is found to be a strikingly conserved feature in other species of *Drosophila*, although the repeats differ in sequence, length and copy number (Gallach, 2014; Menon et al., 2014). It is shown that ectopic expression of hairpin RNA from 1.688^X^ repeats in larvae leads to the generation of abundant siRNAs, increases X recognition by MSL and partially rescues the lethality of *roX1/roX2* mutant males (Menon et al., 2014). Depletion of siRNA-processing endonuclease Dicer-2 (Dcr-2) or siRNA-binding effector protein Argonaute2 (Ago2) has no effect on MSL localization and male survival in the wild-type background; however, it significantly enhances the lethality of *roX1/roX2* mutants (Menon and Meller, 2012; Deshpande and Meller, 2018).

Ectopic integration of 1.688^X^ repeats into the autosome by itself provides some recruitment of intact MSL complexes to the site of insertion and increase of gene expression in the surrounding regions; the observed effects are enhanced by the production of siRNAs (Joshi and Meller, 2017). However, no evidence has been obtained to date that the MSL complex directly interacts with the siRNA-guided RNAi-induced silencing complex (RISC) to increase X-chromosomal gene expression. siRNA pathway function is typically associated with gene repression (Malecova and Morris, 2010). Thus, the mechanism by which 1.688^X^ satellites and their cognate siRNAs contribute to MSL recruitment is still unclear to date. It could be suggested that 1.688^X^ repeats and siRNA-guided RISCs modify the surrounding chromatin regions to ameliorate the spreading of the MSL complex along the X chromosome. 1.688^X^-derived siRNAs in complex with nascent transcripts of euchromatic 1.688^X^ satellite arrays could function as guides attracting chromatin-remodeling factors to regions of active transcription (Joshi and Meller, 2017). However, recent finding of the histone methyltransferase Su(var)3-9 contribution to X recognition (Deshpande and Meller, 2018) does not fit into this hypothesis. Methyltransferase Su(var)3-9 establishes the repressive H3K9me2 mark and takes part in heterochromatin formation. At the same time, only some 1.688^X^ repeats are found to be enriched in H3K9me2 (Deshpande and Meller, 2018). How X recognition and MSL spreading could be assisted by repressive chromatin marks also requires further investigations (Menon and Meller, 2015).

In summary, these findings indicate that siRNAs generated from 1.688^X^ satellite sequences contribute to X localization of the MSL complex. Ago2-siRNA-containing RISCs could presumably bind nascent RNAs and recruit activities that promote a more open chromatin environment on the male X-chromosome, compared to autosomes, to facilitate MSL recruitment.

## Satellites and Meiotic Drive

### Satellites as a Target of Meiotic Drive: Segregation Distorter

Meiotic drive is a meiotic or post-meiotic mechanism causing non-equivalent production of gametes generated by a heterozygous organism, with one type of gametes having an advantage in transmission to offspring. While, according to Mendelian laws of inheritance, homologous chromosomes in meiosis segregate independently, having equal chances of being distributed to gametes, meiotic drive causes a change in allelic frequencies in a population (Crow, 1988). It has far-reaching evolutionary consequences for speciation and also for genome structure. Genetic elements responsible for meiotic drive are currently found in a wide variety of taxa (plants, fungi, insects, and mammals), including the *Drosophila* genus (see Lyttle, 1991; Meiklejohn and Tao, 2010; Lindholm et al., 2016; Courret et al., 2019). The best studied systems of meiotic drive are the *Segregation Distorter* (*SD*) complex in *D. melanogaster* (Sandler et al., 1959; Hartl, 1975; Temin, 1991; Kusano et al., 2003; Larracuente and Presgraves, 2012) and Winters SR system of *D. simulans* (Tao et al., 2007a, b; Lin et al., 2018). The most known meiotic drive systems in *Drosophila* act in males as sperm killers. Meiotic drive generally involves at least two loci: a driver and its target. These loci are tightly linked; they are located in homologous, but opposite chromosomes. During spermatogenesis, the driver disrupts the formation of functional sperm that do not carry the chromosome with the driver itself, acting as selfish element.

**FIGURE 2:**
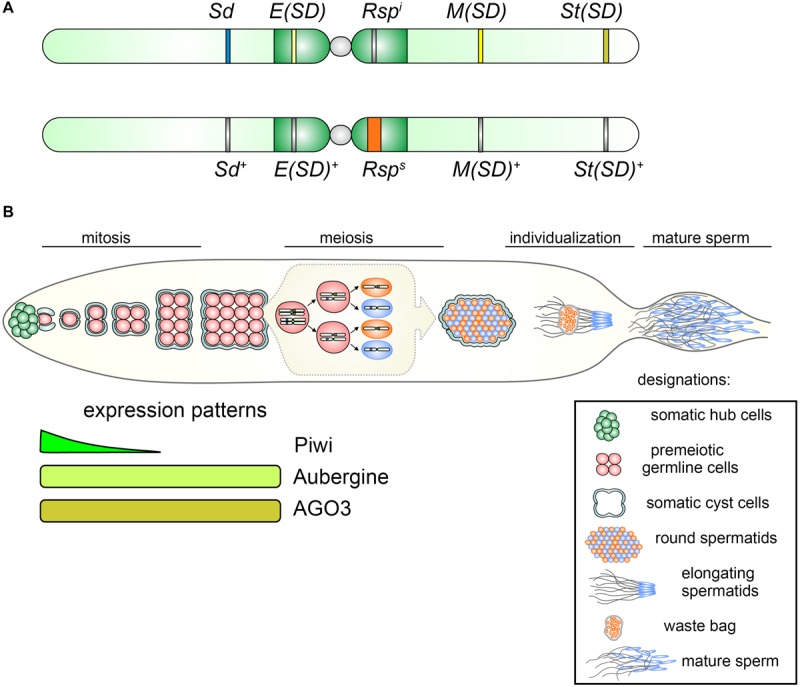
**(A)** Representation of chromosomes 2 of *D. melanogaster* indicating relative positions of components of the SD complex. Top: distorting chromosome 2 carries *Sd, Segregation Distorter; Rsp^i^, Responder i* (insensitive); *E(SD), Enhancer of Segregation Distorter; M(SD), Modifier of Segregation Distorter*; and *St(SD), Stabilizer of Segregation Distorter*. Bottom: non-distorting SD^+^ chromosome 2 contains wild-type loci *Sd*^+^, *E(Sd)*^+^, *M(SD)*^+^, and *St(SD)*^+^ and *Rsp*^s^ (sensitive) allele. **(B)** Top: Overview of SD action during *Drosophila* spermatogenesis. At the apical testis tip (leftward) germline stem cells (red) are located adjacent to the hub (green) and are surrounded by two somatic cyst cells (gray). One of the daughter cells of the germline stem cell, the spermatogonium, undergoes four mitotic divisions to finally create a cyst of 16 spermatocytes. Mature spermatocytes synchronously enter meiosis producing 64 haploid round spermatids. The spermatids nuclei undergo strong condensation, owing to exchanging histones for protamines. The nuclei bearing the *Rsp*^s^ allele (orange) fail to properly condense during the individualization process and are discarded in the waste bag, whereas *Sd*-bearing spermatids (blue) become mature sperm and enter the seminal vesicle, where they are stored until copulation. Bottom: expression patterns of PIWI subfamily proteins, Piwi, Aubergine, and AGO3, in premeiotic germline cells of the testes.

*Segregation distorter* is found with a low frequency (1–5%) in almost all natural populations of *D. melanogaster* (Hiraizumi and Nakazima, 1967; Hartl, 1975; Temin and Marthas, 1984; Temin, 1991; Presgraves et al., 2009; Brand et al., 2015). The main elements of the *SD* complex are the *Sd* locus (*Segregation distorter*) at cytolocation 37D on the left arm of chromosome 2 (the driver) and a block of *AT*-rich complex satellites called *Responder* (*Rsp*), located in the pericentromeric heterochromatin region h39 of the right arm of chromosome 2 (Figure 2A) (Sandler and Hiraizumi, 1960; Hartl, 1973). The *Rsp* locus is the direct target of the action of *Sd*. Being *in trans* to each other, *Sd* and *Rsp* loci interact, causing meiotic drive in male flies, but not in females. In the testes of males bearing the *Sd* locus and a wild-type homologous chromosome 2 (*Sd/Sd* +), most *Sd* + carrying spermatids are not able to pass through the histone-to-protamine transition during spermiogenesis and only *Sd*-carrying spermatids undergo proper genome compaction and individualization and become mature sperm (Figure 2B) (Hartl et al., 1967; Tokuyasu et al., 1977; Kettaneh and Hartl, 1980). The transfer of the *Rsp* block to other chromosome makes it sensitive to the influence of *Sd*, causing a disruption of development of spermatids carrying it (Lyttle, 1989).

### Structure of the Segregation Distorter Complex

The *Sd* locus encodes a truncated from the 3′-end, but functionally active copy of *Ran-GTPase Activating Protein* (*RanGAP*) gene, resulting from a tandem duplication of a 5-kb region of the genome (Powers and Ganetzky, 1991; Merrill et al., 1999; Kusano et al., 2001). The truncated copy (hereinafter *Sd-RanGAP*) is expressed in the testes along with the full-size *RanGAP*, but the Sd-RanGAP protein lacks 234 aa at its carboxyl terminus with the loss of the nuclear export signal and the site of sumoylation. This leads to a partial accumulation of Sd-RanGAP protein in the nucleus, whereas the wild-type protein RanGAP is localized predominantly in the cytoplasm and is enriched on the cytoplasmic side of nuñlear pores (Kusano et al., 2001). Expression of Sd-RanGAP protein is sufficient to induce meiotic drive targeting the sensitive *Rsp* allele *in trans* (McLean et al., 1994; Merrill et al., 1999).

The canonical *Rsp* repeat has a length of 240 bp and consists of two homologous to each other (∼84% identity) left and right halves, each 120 bp in length. In addition to the canonical ones, truncated and diverged copies of *Rsp* repeats are found in the genome, which are only 53–57% identical to the canonical forms (Houtchens and Lyttle, 2003; Larracuente, 2014; Khost et al., 2017). A large and highly variable block of *Rsp* repeats in the pericentromeric heterochromatin of 2R consists of a major locus containing canonical head-to-tail repeats with left and right halves in the region that is directly proximal to the centromere and *Rsp*-like repeats located distally. The block of *Rsp* repeats is interrupted by insertions of transposons at both ends. *Rsp* repeats in the central part of the major locus are highly homogeneous (89.5% identity for the left and 90.4% for the right half), suggesting that gene conversion and non-equivalent crossing over lead to repeated homogenization events through the concerted evolution of the repeats (Larracuente, 2014; Khost et al., 2017).

Sensitivity to the drive correlates with the number of *Rsp* repeats: insensitive alleles, *Rsp*^i^, carry approximately 20–200 copies; sensitive *Rsp*^s^ alleles contain about 700 copies, whereas the highly sensitive *Rsp*^ss^ alleles contain up to 2,500 copies (Wu et al., 1988; Pimpinelli and Dimitri, 1989; Lyttle, 1991; Houtchens and Lyttle, 2003). A minimal number of *Rsp* repeats (less than 20) is located on the same *Sd*-containing chromosome, since in this case the presence of the sensitive form of *Rsp* would leads to the elimination of the chromosome itself (Hartl, 1974). The *Rsp* locus itself is not essential for survival, since its homozygous deletions do not significantly affect the viability and fertility of flies (Ganetzky, 1977).

In addition to *Sd* and *Rsp*, several modifier loci have also been identified that affect the efficiency of the meiotic drive caused by this system (Figure 2A). Among them, located in the right arm of the chromosome 2 at cytolocation 56F *Stabilizer of Segregation Distortion*, *St*(*SD*), is found; the absence of which makes the *SD* effect unstable (Sandler and Hiraizumi, 1960). In the pericentromeric heterochromatin of the left arm of chromosome 2, *Enhancer of Segregation Distortion, E*(*SD*), is located, the presence of which enhances meiotic drive (Ganetzky, 1977). It should be noted that *E(SD)* itself can affect *Rsp* even in the absence of *Sd* locus causing a moderate effect on the sensitive *Rsp* form and a strong effect on the supersensitive chromosome (Sharp et al., 1985; Temin, 1991). In euchromatic region 43E of the right arm of the chromosome 2 *Modifier of Segregation Distortion, M(SD*), is detected, the absence of which suppresses the effect of drive (Hiraizumi et al., 1980). In addition to these positive modifiers, several suppressors of *SD*, *Su(SD)*, are also found on chromosomes 3 and X, whose effects reduce meiotic drive (Trippa and Loverre, 1975; Hiraizumi and Thomas, 1984). It is worth noting, that all these modifiers of *SD* are still not assigned to any defined genes.

### Mechanisms Underlying SD Action

Despite more than 60 years of studies, the molecular mechanisms of the meiotic drive caused by the *SD* complex have not yet been established and this question still puzzles geneticists. Mechanistic disturbances underlying *SD* are not associated with the passage of germ cells through meiosis (Hartl et al., 1967). The earliest manifestation of spermatogenesis defects caused by *SD* action is the failure of chromatin condensation in a half of spermatid nuclei in postmeiotic 64-cell cysts, visible by electronic microscopy (Kettaneh and Hartl, 1976; Tokuyasu et al., 1977; Hauschteck-Jungen and Hartl, 1982). It leads to subsequent defects in spermatid elongation, histone-to-protamine transition and sperm maturation.

The most intriguing questions are associated with the relationship between the processes in which RanGAP is involved in wild-type testes and the status of the *Rsp* locus. Small GTPases of the Ran family are conserved in higher eukaryotes. GTPases Ran and their cofactors RanGAP (Ran GTPase-activating protein) and RanGEF (Ran guanine nucleotide exchange factor) are key components of the nuclear transport of macromolecules. Ran proteins continuously shuttle between the nucleus and the cytoplasm. While RanGAP functions predominantly in the cytoplasm where it is associated with the cytoplasmic side of nuclear pores, RanGEF is located in the nucleus and is associated with chromatin, where it converts Ran-GDP back to Ran-GTP. This circumstance creates a concentration gradient of Ran-GTP and Ran-GDP through the nuclear envelope: Ran-GDP is enriched in the cytoplasm, while Ran-GTP is present in high concentrations in the nucleus (Kalab et al., 2002). This gradient ensures the functioning and a direction of nuclear transport. In addition to their function in nuclear transport, Ran and its cofactors are also necessary for the regulation of mitosis, formation of spindle microtubules and assembly of the nuclear envelope (Sazer and Dasso, 2000; Dasso, 2001; Moore, 2001), but the molecular mechanisms of processes in which Ran is involved beyond nuclear transport, remain obscure. It is shown that a high local concentration of Ran-GTP near chromosomes contributes to the stabilization and assembly of spindle microtubules (Dasso, 2001).

Despite the absence of 234 residues at the C-terminus, Sd-RanGAP can actively function in *in vitro* experiments to stimulate Ran-GTPase activity (Kusano et al., 2001). What is responsible for the *SD* phenotype? Evidently, it is impaired intracellular localization: Sd-RanGAP is found diffusely distributed in the cytoplasm and partly in the nuclei of premeiotic spermatocytes. The presence of enzymatically active Sd-RanGAP inside the nuclei can stimulate premature hydrolysis of Ran-GTP, thus disrupting the formation of Ran-GTP/Ran-GDP gradient across the nuclear envelope. Indeed, the ability of Sd-RanGAP to cause distortion is completely abolished in case of mutations disrupting its enzymatic activity (Kusano et al., 2001). The export of transgenic Sd-RanGAP from the nucleus by attaching a heterologous signal of nuclear export also leads to complete cessation of distortion. Thus, enzymatic activity and impaired localization of Sd-RanGAP are both necessary for meiotic drive.

It should be noted that Sd-RanGAP is expressed in spermatocytes along with the wild-type form of RanGAP, so its effect may be due to a partial disturbance of the normal gradient of Ran-GTP/Ran-GDP. However, overexpression of wild-type RanGAP also leads to the disruption of its intracellular localization and is itself able to cause meiotic drive, like expression of Sd-RanGAP (Kusano et al., 2002). By contrast, increased expression of Ran or RanGEF is found to suppress meiotic drive in any case: expression of *Sd-RanGAP*, over-expression of wild-type *RanGAP* or additional doses of *E(SD)* in *Rsp* sensitive background (Kusano et al., 2001, 2002). It was found that mutations of several nuclear import and export factors enhance distortion, additionally indicating that nuclear transport is essential for segregation distortion (McElroy et al., 2008). Taken together, these data indicate that meiotic drive is caused by disruption of nuclear transport or signal transduction provided by Ran, where the balance of Ran and its cofactors plays a key role.

Analysis of small RNA libraries from the testes reveals the existence of piRNAs mapping to the *Rsp* locus (Nishida et al., 2007; Larracuente and Presgraves, 2012; Gell and Reenan, 2013). However, genomic sources and functions of these piRNAs are unknown. It was found that mutations in *aubergine*, encoding the key component of the piRNA silencing pathway, moderately increase *Sd*-induced meiotic drive (Gell and Reenan, 2013). These data indicate that the piRNA pathway functions in the suppression of distortion. *Rsp*-specific piRNAs could be involved in maintaining the condensed state of *Rsp* repeats, which is necessary for correct spermiogenesis. Several hypothetical models were proposed to explain a possible role of piRNAs in the interaction of *Sd* with *Rsp* (Larracuente and Presgraves, 2012; Gell and Reenan, 2013). According to one of them, Sd-RanGAP-caused disruption of nuclear transport prevents the transfer of piRNA-containing complexes to the nuclei, which is necessary for chromatin remodeling of *Rsp* repeats (Larracuente and Presgraves, 2012). Another hypothesis proposes the disruption of nuclear export, which reduces the availability of precursors for piRNA biogenesis in the cytoplasm (Gell and Reenan, 2013). The authors suggest that *Rsp*-related piRNAs contribute to the epigenetic silencing of the *Rsp* locus via modulation of chromatin states. Disruption of nuclear export of piRNA precursors caused by Sd-RanGAP would lead to the formation of an insufficient number of *Rsp*-piRNA-loaded RNP complexes to maintain effective *Rsp*-silencing, leading to failure in chromatin compaction and subsequent destruction of *Rsp*-carrying spermatids (Gell and Reenan, 2013).

It should be noted that these models are rather not convincing. In premeiotic spermatocytes piRNAs and RISC complexes containing piRNAs and Aubergine or AGO3 proteins normally reside in nuage granules, cytoplasmic perinuclear organelles, where post-transcription silencing of harmful transcripts is carried out (Snee and Macdonald, 2004; Kibanov et al., 2011). The only ARGONAUTE family protein and piRNA pathway participant functioning in the nuclei of germline stem cells and spermatogonial cells is Piwi. Piwi is not expressed in premeiotic spermatocytes and at subsequent spermatogenesis stages, in spermatids (Saito et al., 2006), where *SD* effects are manifested (Figure 2B). Thus, Piwi-containing RISC cannot participate directly in chromatin remodeling and compaction during spermatid development. It could be assumed that piRNAs are involved in the introduction of heterochromatic epigenetic marks during earlier stages of spermatogenesis, and these marks are maintained throughout germ cell divisions including meiosis. However, experimental evidence for this hypothesis is not presented to date. Taking into account the moderate impact of piRNA silencing disorders on meiotic drive caused by the *SD* complex, piRNAs may play only an auxiliary functional role in the mechanism of segregation distortion prevention. It can be proposed that future investigations including genome- and transcriptome-wide approaches and deep analysis of *Rsp*-related small RNAs will shed a light on the underlying mechanisms of satellite-mediated segregation distortion.

## Contribution of Satellite Repeats to Reproductive Isolation

Previously published studies demonstrate that satellites are one of the most rapidly evolving parts of the genome even in closely related species exhibiting considerable differences in their content and genomic distribution. Despite the known loci involved in hybrid incompatibility and reproductive isolation mainly being protein-coding genes (Barbash and Ashburner, 2003; Presgraves et al., 2003; Brideau et al., 2006; Kotov et al., 2019), fast satellite expansion and divergence also could also contribute to reproductive isolation and speciation, creating a specific genetic background in interspecies hybrids and causing hybrid incompatibilities. The underlying mechanisms may include modulation of chromatin compaction by satellite arrays, disruption of chromosome pairing in mitosis or meiosis and participation of satellites in meiotic drive.

**FIGURE 3:**
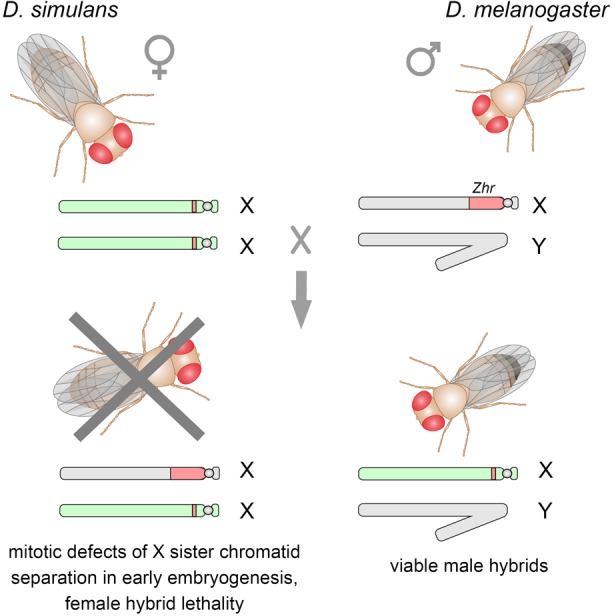
Crosses between *D. simulans* females and *D. melanogaster* males produce viable F1 male hybrids, whereas F1 hybrid females die as embryos. The X-linked *Zhr* locus containing a tremendous 359-bp satellite block causes hybrid lethality by inducing chromosomal segregation defects in early embryos owing the failure of the 359-bp satellites to maintain proper heterochromatin structure. Sex chromosomes inherited from *D. melanogaster* are shown in gray, from *D. simulans* – in light green. *D. melanogaster Zhr* locus marked by red, as well as a small block of the 360-bp repeat variant in the pericentromeric region of *D. simulans* X chromosome.

It is known that closely related species *D. melanogaster* and *D. simulans* are completely reproductively isolated (Sturtevant, 1920; Davis et al., 1996). They possess considerable differences in the heterochromatic regions of their genomes including distribution and composition of satellites (Strachan et al., 1985; Lohe and Roberts, 1988; Jagannathan et al., 2017). Sawamura and coworkers found a direct quantitative link between the presence of an X-linked locus, initially called *Zygotic hybrid rescue* (*Zhr*), in the *D. melanogaster* genome and female hybrid lethality in crosses of *D. simulans* mothers and *D. melanogaster* fathers (Sawamura and Yamamoto, 1993; Sawamura et al., 1993). This region mainly consists of a tremendous block of 359-bp 1.688 satellites and is located in pericentric heterochromatin of the X chromosome (Figure 3). In the *D. simulans* genome only a small block of divergent related 360-bp satellites is detected in X pericentric heterochromatin (Strachan et al., 1985; Lohe and Roberts, 1988; Kuhn et al., 2012). Subsequently it was found that hybrid females died in early embryogenesis due to mitotic defects in X sister chromatid separation (Ferree and Barbash, 2009). It was shown that the presence of the 359-bp satellite megablock in the hybrid embryos induces asynchrony of divisions and segregation failures of the paternally inherited X^mel^ chromosome during mitotic cycles 10–13 of the syncytial blastoderm stage (Figure 3). Despite their proper condensation in prometaphase, X^mel^-linked pericentric 359-bp satellites exhibit abnormal stretching, generation of chromosome laggings, and aberrant enrichment by Topoisomerase 2 during mitotic anaphase (Ferree and Barbash, 2009). Expectedly, the hybrid male embryos carrying only the maternal X^sim^ chromosome undergo normal nuclear divisions at the syncytial blastoderm stage, subsequent passage through gastrulation and survival to adulthood (Sturtevant, 1920; Ferree and Barbash, 2009). Since hybrid females from reciprocal crosses are viable (Barbash and Ashburner, 2003), one can suggests that unknown maternal factors are strongly required for heterochromatin formation of this species-specific satellite block. The putative mechanisms can involve divergent DNA-specific satellite-binding proteins, as well as satellite-derived small RNAs functioning in heterochromatin packaging (Ferree and Prasad, 2012). This is a spectacular example of how differences in non-coding sequences between species directly lead to reproductive isolation owing to defects in mitotic chromosome segregation.

Another crucial process that could putatively be affected by satellite divergence in interspecies hybrids is homolog pairing in meiosis. However, despite the divergence in satellite content of pericentromeric regions, interspecies homologous chromosome pairing and segregation appear to occur normally during male meiosis (Yamamoto, 1979). Note that recombination does not take place in male meiosis and occurs only in the female germline. Additional experiments are required to discern the contribution of pericentromeric satellites to homolog pairing in hybrid females (Ferree and Prasad, 2012). Recent results indicate, however, that even large differences in abundance of the (AATAT)_n_ satellite in pericentromeric chromatin of the chromosome 4 between *D. simulans* and *D. melanogaster* do not cause defects in chromosome segregation in female meiosis (Gilliland et al., 2015).

## Evolution of Satellite DNAs

As a rule, satellites are divergent both within a species and in the genomes of closely related species, indicating their rapid evolution (Plohl et al., 2012; Wei et al., 2014; Garrido-Ramos, 2017; Jagannathan et al., 2017; Lower et al., 2018). A characteristic feature of the evolution of satellites is the considerable variability in the number of repeats along with the relative conservation of repeating units. In addition to rapidly evolving satellites, there are extremely conservative satellite DNAs considered “frozen.” For example, the *dodeca* satellite is found in such distal species as *D. melanogaster*, *Arabidopsis thaliana*, and *Homo sapiens* (Abad et al., 1992) that indicates strong stabilizing selection. In some cases, satellites can be maintained at low copy number in the common ancestor and then be greatly increased in copy number in the daughter species, leading to a common set of satellite DNAs in the related taxa (the so-called library hypothesis), with different variants prevailing in different species (Salser et al., 1976; Fry and Salser, 1977; Lohe and Brutlag, 1987; Mestrović et al., 1998; Palomeque and Lorite, 2008; Cafasso and Chinali, 2014; Mehrotra and Goyal, 2014). According to the library hypothesis, the emergence of new satellites appears to represent amplification of one of the satellites already present in the ‘library” of short tandem arrays rather than their *de novo* appearance (Fry and Salser, 1977).

The formation of new repeats begins as a result of a duplication of a genomic region (Ruiz-Ruano et al., 2016). Generally homologous recombination generates repeated sequences from single-copy sequences only as rare events, but it can generate duplications from preexisting repeats. Short tandem duplications may arise due to polymerase replication slippage. The replication slippage occurs due to a local denaturation of DNA strands and DNA polymerase premature pausing during replication of a repeating DNA followed by the renaturation of the strands with displacement and reassembly of the replication complex, resulting in a twice replicated DNA region (Levinson and Gutman, 1987; Charlesworth et al., 1994; Viguera et al., 2001). Relatively long duplications arise due to unequal crossing-over (Smith, 1976), rolling circle replication (Cohen et al., 2003; Cohen and Segal, 2009), or repeated insertions of transposable elements at the same site (Meštrović et al., 2015). The non-autonomous transposon *Tetris* was discovered in the genomes of *D. virilis* and *D. americana*. *Tetris* possess terminal inverted repeats including an intermediate outer domain with a variable number of 220-nt tandem repeats (TIR-220). During the analysis of *D. virilis* genome assembly TIR-220 repeats were found as long and homogeneous tandemly repeated DNA arrays up to 66 copies accumulated close to the heterochromatin of the chromosome 2 (Dias et al., 2014). The authors showed that TIR-220 repeats underwent tandem amplification inside *Tetris*. Presumably, the amplification of TIR-220 may cause *Tetris* disruption thus facilitating the generation and expansion of satellite arrays (Dias et al., 2014). Recent analysis of the genomes from several populations of *D. melanogaster* allowed to reveal that transposons of three major types [Long Terminal Repeats (LTR) retrotransposons, non-LTR retrotransposons, and DNA transposons] often form tandem dimers owing to insertion site preference during periods of their active transposition (McGurk and Barbash, 2018). The authors postulate three mechanisms of tandem dimer formation from transposable elements, including ectopic recombination between long terminal repeats of LTR retrotransposons; circularization and rolling circle replication of transposon with subsequent insertion of the resulting concatemer; double insertions of a transposable element at the same target site. Thus abundant dimers of transposable elements potentially can expand providing building blocks for subsequent transformation into satellite arrays. Expansion events were observed for DNA transposon *hobo* estimated to have 13–19 tandem copies in one of the analyzed strains (McGurk and Barbash, 2018). Transposable elements appear to significantly contribute to satellite evolution by generating libraries of tandem repeats and, in addition, the movement of transposable elements together with satellite DNAs can be a mechanism for distribution of the latter throughout the genomes of multiple eukaryotic organisms (Meštrović et al., 2015).

Duplicated regions may undergo amplification due to unequal crossing-over forming large clusters (Stephan and Cho, 1994). Subsequently each cluster can evolve independently with changes in the number of repeating elements or their sequence. Unequal crossing-over or gene conversion mechanisms give advantage to the most common types of repeating element within a cluster, contributing to homogenization of satellite sequences. However, sometimes mutations arising in one of the repeats can be spread throughout the entire cluster due to the same mechanisms, leading to the formation of a new family of satellite DNA. This special mechanism of evolution, including mutation, homogenization of the new sequence and subsequent fixation in the population, is called concerted evolution (Dover, 1982, 1986; Liao, 1999). A characteristic low sequence variability between satellite monomers appears to be the sum of two oppositely directed processes: the acquisition and accumulation of nucleotide substitutions as well as their rapid spreading or disappearance in the result of non-reciprocal sequence transfer by unequal crossing-over, gene conversion, rolling circle replication, transposition, and other unknown mechanisms (Plohl et al., 2012). According to this model, satellite mutations accumulate and gradually spread within species, as is shown for satellite family *ATOC180* that is specific for closely related species *Drosophila obscura, Drosophila ambigua*, and *Drosophila tristis* (Bachmann and Sperlich, 1993). Concerted evolution causes the divergence of common ancestral satellite sequences in closely related but reproductively isolated species with high sequence homogeneity in each of them as it is found for satellites of the 1.688 family in *D. melanogaster* and five other species from the *melanogaster* group (Kuhn et al., 2012).

Evolution of centromere satellites is a subject of particular interest. Since centromere functions are crucial for normal chromosome segregation during cell division, it can be expected that centromeric satellite sequences and centromere-specific proteins are conserved; however, both centromeric DNAs and proteins rapidly evolve. This contradiction is called the “centromere paradox” (Henikoff et al., 2001; Malik and Bayes, 2006). The spindle of a cell-precursor in the female meiotic division is oriented such that one of each pair of homologous chromosomes is transferred into the oocyte and the other to the polar body with equal probability. However, it is shown that centromeres with expanded satellite repeats cause the centromere drive in *Mimulus* monkeyflowers and in mice (Fishman and Saunders, 2008; Akera et al., 2017, 2019; Iwata-Otsubo et al., 2017). The expansion of satellite repeats leads to a greater likelihood of transfer of the corresponding chromosome to the oocyte; such centromeres are called “strong.” “Strong” centromeres contain more satellite repeats; they form more CENP-A nucleosomes for kinetochore assembly and bind more kinetochore proteins (CENP-C and HEC1) relative to the “weaker” centromeres (Iwata-Otsubo et al., 2017). This asymmetry of the meiotic spindle of division is established owing to the presence of CDC42 signaling and RAN-GTPase gradient, which cause the cortical polarization and subsequent migration of one spindle to the cortex in the site of the formation of the polar body, and to post-translational modification (tyrosination) of microtubule α-tubulin (Akera et al., 2017; Lampson and Black, 2017; Kursel and Malik, 2018). The second spindle of division remains in the center of the cell and does not undergo the modification. “Strong” centromeres more easily detach from tyrosinylated microtubules and have a greater affinity for unmodified ones that ultimately leads to the preferred orientation of the corresponding chromosomes toward the developing oocyte. The reasons for this difference in affinity is not clear now, but it can be assumed that an increase in the number of satellite repeats leads to a greater binding of centromere-associated proteins, such as Aurora B kinase and MCAK (mitotic centromere-associated kinesin) (Peris et al., 2009; Akera et al., 2019). These proteins are responsible for microtubule depolymerization, and tyrosinylated microtubules can be a preferred substrate for them.

According to the centromere drive hypothesis, satellite expansion firstly leads to the generation of a centromere with enhanced microtubule binding abilities. Centromeric satellite DNAs can also evolve, changing the affinity for kinetochore proteins and oocyte-oriented spindle microtubules for increasing the likelihood of transmission of the chromosomes carrying them to the oocyte. Thus, centromeric satellite DNAs in the female germline can function as selfish genetic elements, promoting preferential transmission to progeny of one chromosome compared to its homolog during the asymmetrical meiotic process. However, this can reduce the viability of the offspring, because the chromosomes that benefit from the drive may carry linked deleterious mutations. At the next stage, evolutionary changes in CENP-A can lead to stronger affinity to a weaker centromere to reduce the negative effects associated with the centromere drive. Indeed, in *D. melanogaster*, CENP-A (CID) protein evolves rapidly under positive selection (Malik and Henikoff, 2001). The corresponding CID mutations expectedly become fixed in the population (Henikoff and Malik, 2002; Malik, 2009; Rosin and Mellone, 2017). This, in turn, triggers a new round of changes in the centromeric satellite DNAs. Thus, the existing co-evolution leads to an “arms race” between centromere DNA sequences and centromere proteins, despite their conserved and vital functions.

## Conclusion and Perspectives

**FIGURE 4:**
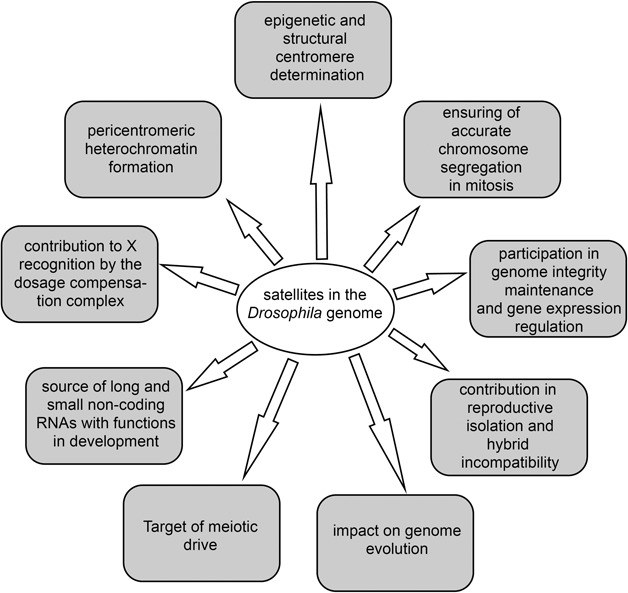
The main functions of satellites in the *Drosophila* genome.

Satellite repeats represent one of the most mysterious components of the genomes. Because initially, protein-coding functions were not attributed to the satellites, they were earlier considered as useless DNA, that are parasitically expanding in the genomes. Multiple data obtained to date allow to shed light upon the important properties of satellites in the eukaryotic genome. *D. melanogaster* is one of a few species where satellite DNAs have been comprehensively mapped in the genome. Thus, *D. melanogaster* is an excellent model organism for studying satellite DNAs, including the discovery of novel functions of satellites that appear to be relevant for a wide range of eukaryotic organisms (Figure 4). Here we focused on recent findings of satellite DNA studies in the genomes of *Drosophila* obtained by using as classical genetic and molecular biology methods as well as new technological approaches and bioinformatics. Advanced genome assembly methods allowed resolving the organization of functional centromeres of *D. melanogaster* in detail. Centromeres are composed of retrotransposons intermingled with large blocks of satellite repeats. Satellite arrays within centromeres can exhibit the formation of secondary DNA structures such as dimeric i-motifs, which facilitate centromere identification. Thus, centromere formation may be based on both structural motifs in centromeric DNA and epigenetic mechanisms functioning in concert. Satellite arrays are basic structural platforms for centromeric and pericentromeric regions of the genome; they play significant roles in heterochromatin formation, dosage compensation, reproductive isolation, genome stability, and evolution. Satellite DNAs are maintained through natural selection because of their importance in the preservation of vital regions of the genome. They have a unique evolutionary mechanism of concerted evolution, which includes processes of nucleotide substitutions in the precursor sequence with subsequent homogenization throughout monomers of repetitive arrays and fixation within an individual species. Many satellites are transcribed with the generation of long or small non-coding RNAs, and misregulation of their expression causes various defects in the maintenance of genomic architecture and compaction, chromosome segregation, larval and pupal viability and gametogenesis, although the underlying molecular mechanisms often remain elusive. Some centromeric repeats are transcriptionally active during mitosis and their transcripts are essential for promoting kinetochore stabilization and centromere cohesion. It is known that satellite misregulation is associated with a number of human diseases, such as hereditary diseases, developmental abnormalities, different cancers, and neurodegenerative disorders. Chromosomal fragility can have relations to defects in the maintenance of satellite loci (Black and Giunta, 2018). Changes in transcription of satellite DNAs can also be associated with aging (Ferreira et al., 2015; Smurova and De Wulf, 2018; Zhu et al., 2018) and cancer progression (Bersani et al., 2015). Malignant cell transformations are found to be linked with epigenetic abnormalities in satellite loci (Ehrlich et al., 2003; Bruckmann et al., 2018). Analysis of satellite DNAs or their transcription can be used for diagnostic purposes in the detection of pathologies and for drug development (Kondratova et al., 2014; Kishikawa et al., 2016). We propose that studies in *Drosophila* provide a better understanding of satellite contribution to the genome organization, evolution, and association with developmental defects and disease.

## Author Contributions

AS and LO prepared the initial version of the manuscript. AK, SB, and VA rigorously revised and improved the manuscript. VA and LO created figures and tables. AS, LO, and AK polished the final version of the manuscript. All authors provided intellectual contribution, edited, and approved the manuscript for publication in its present version.

## Conflict of Interest

The authors declare that the research was conducted in the absence of any commercial or financial relationships that could be construed as a potential conflict of interest.
